# Urothelial carcinoma of donor origin in a kidney transplant patient

**DOI:** 10.1186/s40425-016-0167-4

**Published:** 2016-10-18

**Authors:** Rosa M. Michel Ortega, Daynna J. Wolff, Cynthia A. Schandl, Harry A. Drabkin

**Affiliations:** 1 Division of Hematology and Oncology, Medical University of South Carolina, 173 Ashley Ave, Suite 102 BSB, Charleston, SC 29425 USA; 2 Department of Pathology & Laboratory Medicine, Medical University of South Carolina, Charleston, SC USA

**Keywords:** Post-transplant malignancy, Immunosuppression, Solid organ transplantation, Urothelial carcinoma

## Abstract

**Background:**

Malignancy after transplantation is an uncommon multifactorial occurrence. Immunosuppression to prevent graft rejection is described as a major risk factor in malignancy development in the post-transplant state. Donor-derived malignancy is a rare reported complication. Herein, we review our patient history and discuss diagnostic strategies and the implications of immunosuppression for donor-derived malignancy.

**Case presentation:**

This is a 69-year-old man with post-renal-transplant urothelial carcinoma determined to be of donor origin. His course was complicated by BK virus at six years post-transplant; urothelial carcinoma was identified nine years post-transplant. Cystectomy was performed, but because of immunosuppression and underlying chronic kidney disease, the patient was considered ineligible for adjuvant chemotherapy. Two years after resection, screening MRI demonstrated retroperitoneal lymphadenopathy and a right upper pole mass in the transplanted kidney. Urine cytology confirmed the presence of malignant cells; FISH showed 2-8 copies of the X chromosome and no Y chromosome consistent with female origin of the malignant cells. CT-guided renal mass and paraaortic lymph node biopsies demonstrated that about 50 % of cells had an XY complement, while the remainder showed a XX genotype by chromosomal SNP microarray analysis. Immunosuppression was discontinued and the donor kidney removed. X/Y FISH of the urothelial carcinoma identified in the explanted kidney confirmed that the malignant cells were of female donor origin. Follow-up at 3, 6 and 12 months after discontinuation of immunosuppression and surgery demonstrated normalization of the lymphadenopathy and absence of new lesions.

**Conclusions:**

Immunosuppression is a major risk factor for development of malignancy in transplant recipients. Donor-derived malignancy can arise and current molecular studies allow an accurate diagnosis. Withdrawal of immunosuppression and surgical resection of the transplant kidney proved an effective treatment in our case.

## Background

According to the Organ Procurement and Transplantation Network (OPTN), there were 17,878 kidney transplants in 2015 in the United States with 5628 of them obtained from living-donors [1]. In order for the graft to survive in the host, the recipient’s immune response to alloantigens from the graft is modulated. Otherwise, rejection would cause tissue damage and graft organ failure. Immunosuppressive drugs have made such immunomodulation feasible with 1-year graft-survival rates of 80 to 90 % [2]. However, such treatment comes at the expense of increased incidence of infection and malignancy. The most common cancers in this scenario are non-melanoma skin cancer, non-Hodgkin lymphoma and lung, liver and kidney in recipients of those organs [3]. Development of malignancy is multifactorial in this context. Commonly cited etiologies for new malignancy include decreased immunosurveillance secondary to immunosuppressive drugs, infections with oncogenic viruses, and other host-specific risk factors such as age, comorbidities and smoking and alcohol use. The risk of donor-derived transmission of disease is generally considered negligible [4].

Immunosuppression controls not only anti-graft but also anti-cancer immunity, and as such is most commonly implicated while the specific immunosuppression regimen is less important. A 2010 clinical trial of cadaveric kidney transplant recipients with a 20-year follow-up period randomly allocated patients to azathioprine and prednisolone, cyclosporine monotherapy, or cyclosporine monotherapy followed by azathioprine and prednisolone after the first 3 months of post-transplant. No specific immunosuppressive drug combination was more detrimental than another [5]. Instead, factors such as increasing age and smoking status were associated with increased risk of malignancy in a multivariate analysis. This study however, did not include patients exposed to newer calcineurin inhibitors, such as tacrolimus, the mammalian target of rapamycin (mTOR) inhibitor, sirolimus or the antiproliferative agent mycophenolate mofetil.

We report on a patient that was treated with decreased immunosuppression and surgical removal of transplant kidney for management of donor-derived high-grade urothelial carcinoma.

## Case presentation

A 69-year-old Caucasian male with a past medical history of hypertension and end-stage renal disease (ESRD) secondary to IgA nephropathy was managed with peritoneal dialysis (PD) for 1.5 years. His sister consented to provide a kidney and transplantation was performed in 2004. His post-transplant course was uncomplicated and immunosuppression was maintained with tacrolimus and sirolimus. In October 2010, he developed BK viremia and nephropathy, which was treated with leflunomide and a decrease in the tacrolimus dose. His course was further complicated by multiple cutaneous squamous carcinomas and a cutaneous basal cell carcinoma treated with Mohs surgery. In February 2013, he developed gross hematuria, which prompted further work-up. Masses identified by cystoscopy were sampled by transurethral resection and pathology showed high-grade invasive urothelial carcinoma of the bladder. In April 2013, he underwent radical cystectomy, prostatectomy and left pelvic lymphadenectomy (pT3pN0) with the discovery of an incidental adenocarcinoma of the prostate (Gleason 3 + 3 pT2c). Both native kidneys and ureters were removed and an ileal conduit created. He did not receive adjuvant chemotherapy because of the immunosuppression (prednisone, tacrolimus and sirolimus, which was later switched to mycophenolate mofetil) and chronic kidney disease.

**Fig. 1:**
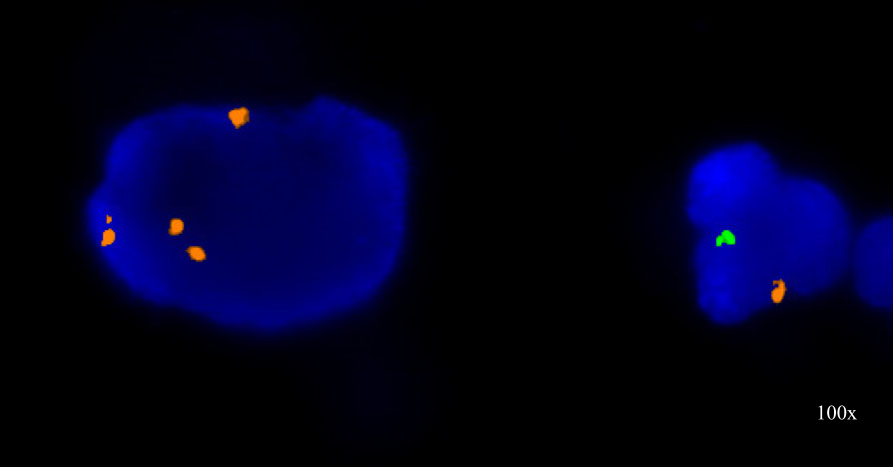
FISH on the urine cytology sample with probes for the X chromosome centromere (*red*) and the Y chromosome heterochromatic region (*green*) (Abbott Molecular, Downers Grove, IL); large malignant urothelial carcinoma cell with four X chromosome signals and a normal male cells with one X and one Y chromosome signal

He was followed with active surveillance and imaging every 6 months. In March 2015, MRI demonstrated extensive retroperitoneal abdominal and pelvic adenopathy and a mass in the right upper pole of the transplant kidney while the patient was completely asymptomatic and kidney function unchanged. Urine cytology was positive for malignant cells that were shown to be of female origin by fluorescence in situ hybridization (FISH) with 2–8 copies of the X chromosome and no copy of the Y chromosome (Fig. 1). CT-guided biopsies of the kidney mass and a lymph node were positive for urothelial cancer. SNP microarray-based chromosome analysis of the lymph node using the I Scan® System with Infinium® OExPls CytoConsortium Array BeadChip (Illumina, Inc, San Diego, CA) demonstrated about 50 % of cells had an XY complement, while the remainder showed a XX genotype. Additional chromosomal aberrations included amplification of regions of 1q, 6p, and 10p and loss of 8p, findings that are consistent with the diagnosis of urothelial cancer. However, the origin of the cancer by single nucleotide polymorphism (SNP) analysis could not be assigned due to equal percentage of the genotypes determined by equal sex chromosome distribution as mentioned above. Pathology and FISH were compared to his original high-grade urothelial carcinoma, which demonstrated aberrations common to urothelial cancer, but not identical to the more recent sample indicating a new clonal process.

**Fig. 2:**
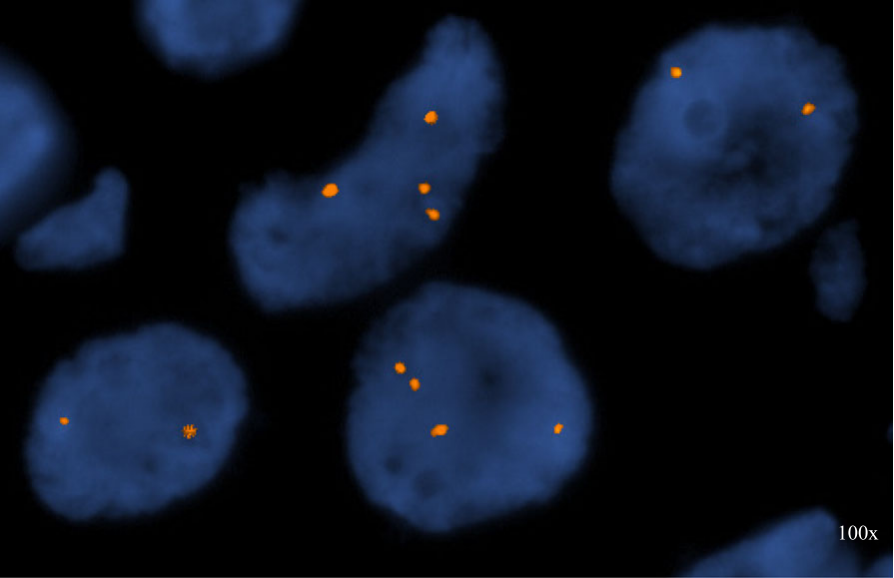
FISH on the surgical specimen post resection with probes for X chromosome centromere (*red*) and the Y chromosome heterochromatic region (*green*) (Abbott Molecular, Downers Grove, IL) showing only X chromosome signals consistent with female donor origin of the urothelial cancer

In June 2015, he underwent transplant nephrectomy, peritoneal dialysis catheter placement, excision of the ileal conduit and parastomal hernia repair. Pathology of the explant demonstrated a 4.6 × 4.4 × 4.1 cm, high grade urothelial carcinoma (pT3) with lymphovascular invasion in the background of severe chronic glomerulosclerosis. The malignant cells were CK7 positive, CD10 partially positive, and CD34 negative. FISH for the X and Y chromosomes performed on the urothelial carcinoma from the explanted transplant kidney confirmed that the malignant cells were of female donor origin (Fig. 2). All immunosuppression was discontinued and PD was resumed. Initial follow-up scans at 3 months demonstrated no new sites of metastasis and decrease in the size of retroperitoneal lymph nodes (LNs) with the index LN shrinking from 1.9 to 0.8 cm. Repeat scans after an additional 3 months also found no new sites of metastasis and further reduction of the index LN size to 0.7 cm. The most recent scans done 1 year after initial diagnosis continue to show no evidence of disease and stability of the index LN size at 0.7 cm. He has resumed all regular activities and has a completely normal ECOG (Eastern Cooperative Oncology Group) performance status (i.e., ECOG = 0).

## Discussion

In the era where therapeutic reactivation of the immune system against cancer has gained significant momentum, this case points out how surgical removal of the primary donor-derived malignancy and tapering of immunosuppression to allow the body to mount a response proves to be effective therapy for at least 1 year follow-up. It is also the first documented case known to the authors to demonstrate donor-derived origin in post-transplant urothelial carcinoma by FISH.

Reduction of immunosuppression, the initial intervention provided to this patient, has been previously described in the management of aggressive squamous cell carcinomas [6] and aggressive undifferentiated epithelioid tumor [7] in kidney transplant recipients. Unlike these tumor types, renal cell and urinary tract carcinoma incidence did not vary significantly during the functional life of the transplant versus after transplant failure and subsequent decreased immunosuppression based on retrospective data from the Australia and New Zealand Dialysis and Transplant Registry [8].

Our patient additionally underwent removal of the graft after prolonged discussion of preferred renal replacement therapy and risks and benefits of a surgical approach were addressed.

However, decrease and/or modification of immunosuppression and, ultimately, removal of the primary malignancy, if arising in the transplanted organ, might not always be feasible as patients might be reluctant to return to dialysis.

He had a history of prior complications associated with an immunosuppressed state including BK viremia in 2010 and urothelial carcinoma that warranted surgical resection in 2013.

The oncogenic potential of BK virus remains controversial [9]. BK virus is considered to cause a subclinical primary infection, and then establishes a latent infection in the kidney and urinary tract, amongst other tissues. When it reactivates, it can cause hemorrhagic cystitis, ureteric stenosis and nephritis. It has also been associated with the development of bladder and kidney cancer, as its viral sequence and T antigen (TAg) has been detected in urothelial carcinoma cells [10]. TAg binds and inactivates p53 and pRb, resulting in aberrant cell cycle regulation [11, 12]. In contrast, Rollison et al. concluded that BK virus did not play a major role in the pathogenesis of bladder carcinoma, as only 5.5 % of the bladder cancer samples of 76 patients with urothelial carcinoma were BK positive by polymerase chain reaction (PCR) and none of them showed TAg expression. These cases were not specifically post-transplant, however, and immune state was not reported [13]. It is beyond our scope to postulate if the prior BK viremia had any impact on the development of donor-derived urothelial carcinoma in our patient. However, he did not have any other known risk factors [14] as he had only been a smoker for 2 years, less than half a pack per day- and had quit more than 40 years prior to the malignancy diagnosis.

The transmission frequency of malignancy by donors, deceased or living, is considered to be less than 1 % [4, 15]. Living donors are regularly screened and in “good health”, consequently diagnosis is frequently made only after donation. Of note, these cancers are considered donor-derived and not donor-transmitted, as the cancer was technically derived from the donor cells, but not clinically present at the time of transplant [16]. There have been at least two case reports of donor-derived urothelial carcinoma in kidney transplant recipients. Both received deceased donor kidneys, had malignancy and metastatic disease diagnosed within 1 year of transplantation, and management included paclitaxel-based chemotherapy. In the first patient, diagnosis of donor-derived malignancy was construed secondary to tempo of disease, lack of native urothelial involvement and inconclusiveness of histocompatibility testing of the tumor; this patient demonstrated no signs of disease over the 3 year follow-up period after halting immunosuppression, removal of the transplanted kidney and chemotherapy [17]. The second patient received the same treatments, but unfortunately passed away from parietal hemorrhage during chemotherapy; interestingly, the liver recipient from the same donor was also found to have disease, biopsy-proven malignant nodules, once the state organ procurement was advised of the neoplasm transmission, whereas the recipient of the other kidney did not [18]. In contrast, our patient developed donor-derived malignancy 11 years after transplantation from a living-related donor and had a sustained response 1 year after stopping immunosuppression and removal of the transplant with no chemotherapy with no current evidence of disease.

Once the clinical suspicion is present, assessment of molecular features of the tumor and/or the presence of XY or XX chromosomes discordant with the recipient’s sex, as in our case, enable the diagnosis. Male donor-derived cells in the basal layer and invasive areas of squamous cell carcinomas of three female kidney transplant recipients has been previously published [19]; however, to our knowledge; this is the first case of high grade urothelial carcinoma of donor-derived origin which is confirmed by FISH. No studies for the presence of BK virus were performed on the explant as management would not be affected.

## Conclusions

Immunosuppression is a major risk factor for development of malignancy in transplant recipients. Donor-derived malignancy can arise and current molecular studies allow an accurate diagnosis. Withdrawal of immunosuppression and surgical resection of the transplant kidney proved an effective treatment with ongoing surveillance and the caveat of return to PD for ESRD management. Continuous communication amongst treatment teams and the patient allowed a good outcome.

## Abbreviations

CKD: Chronic kidney disease
ECOG: Eastern Cooperative Oncology Group
ESRD: End stage renal disease
FISH: Fluorescence in situ hybridization
LNs: Lymph nodes
mTOR: mammalian target of rapamycin
OPTN: Organ Procurement and Transplantation Network
PCR: Polymerase chain reaction
PD: Peritoneal dialysis
SNP: Single nucleotide polymorphism
TAg: T antigen
